# The mediating role of anxiety and depression in the relationship between alexithymia, somatosensory amplification, and functional impairment in fibromyalgia

**DOI:** 10.3389/fpsyt.2025.1598901

**Published:** 2025-07-09

**Authors:** Arda Kazim Demirkan, Gizem Gerdan

**Affiliations:** ^1^ Department of Psychiatry, Samsun Liv Hospital, Samsun, Türkiye; ^2^ Department of Psychology, Division of Clinical Psychology, Izmir Democracy University, Izmir, Türkiye

**Keywords:** Fibromyalgia Syndrome, anxiety, depression, alexithymia, somatosensory amplification, functional impairment

## Abstract

**Objective:**

Fibromyalgia Syndrome (FMS) is a chronic multifaceted condition characterized by widespread musculoskeletal pain, fatigue, cognitive difficulties, and emotional distress, predominantly affecting women. Although psychological factors are frequently implicated, their interrelations remain unclear. Key variables include alexithymia (particularly the difficulty identifying feelings [DIF] subdimension), somatosensory amplification (SSA), and mood symptoms. This study aimed to examine differences in alexithymia, anxiety, depression, and SSA between individuals with FMS and healthy controls, and explore how these variables relate within the FMS group.

**Methods:**

The study included 283 women (mean age = 31.84, SD = 4.02), comprising 142 FMS patients (mean age = 32.20, SD = 4.41) and 141 healthy controls (mean age = 31.48, SD = 3.58). Participants completed self-report measures assessing alexithymia, anxiety, depression, SSA, and functional impairment. Statistical analyses included independent samples t-tests and multivariate analyses of covariance (MANCOVA) to compare groups, and mediation analyses to examine indirect effects of anxiety and depression.

**Results:**

The FMS group reported significantly higher levels of anxiety, depression, DIF, and SSA compared to controls. However, after controlling for anxiety and depression, DIF differences were attenuated and SSA differences were no longer statistically significant. Within the FMS group, individuals with high alexithymic traits also exhibited higher SSA and mood symptoms; however, SSA elevations were no longer evident after accounting for anxiety and depression. Mediation analyses revealed that anxiety and depression significantly mediated the relationship between (a) DIF and functional impairment, and (b) SSA and functional impairment.

**Conclusion:**

Findings underscore the importance of emotional dysregulation and somatic sensitivity in FMS. Anxiety and depression appear to be key pathways linking these psychological traits to functional outcomes. Interventions aimed at improving emotional awareness and regulation may alleviate mood symptoms and enhance daily functioning in individuals with FMS.

## 1 Introduction

Fibromyalgia Syndrome (FMS) is a chronic and multifactorial condition characterized by pain in multiple spinal segments, as well as by allodynia (pain resulting from stimuli that do not normally provoke pain) and hyperalgesia (increased response to painful stimuli), often in the absence of identifiable tissue damage (1). It is widely regarded as a central sensitization syndrome (2), in which the central nervous system amplifies sensory input (3). Symptoms are accompanied by fatigue, cognitive disturbances, and sleep problems, typically without identifiable medical explanation (4). The global prevalence of FMS ranges between 0.5% to 5.0% (5–7), disproportionately affecting women (8, 9). This condition significantly impairs quality of life (10), increases healthcare utilization, and contributes to loss of productivity (11). Despite its disabling nature, the etiology and pathophysiology of FMS remain elusive, and it is often categorized as a functional somatic syndrome or medically unexplained condition due to the absence of consistent biomarkers (12–14).

Diagnostic criteria for FMS have evolved over time, with the American College of Rheumatology (ACR) first establishing tender point-based criteria in 1990, later revised in 2010 and 2016 to incorporate widespread pain index (WPI), symptom severity (SS) scales, and exclusion of other disorders (4, 15). These revisions reflect growing recognition of FMS as a multidimensional syndrome, not solely defined by musculoskeletal pain but also by cognitive dysfunction, fatigue, unrefreshing sleep, and affective symptoms.

Despite the widespread prevalence of FMS, its impact on psychosocial functioning, and its financial cost to society, several factors contribute to its misunderstanding among clinicians, often leading it to be labeled as a psychosomatic condition with negative connotations. These factors include its unclear etiology, its frequent coexistence with other psychiatric diseases, and patients’ regular outpatient clinic visits with a variety of symptoms that can’t be attributed to a specific disease (16). Although the majority of patients report various somatic and musculoskeletal complaints, the frequently normal results of routine blood tests and radiological examinations seem to support the consideration of the condition as psychosomatic (17).

Somatization refers to the process by which psychological distress is expressed through physical symptoms, often in the absence of identifiable medical pathology. This phenomenon is central to the understanding of many functional somatic syndromes, including FMS, and plays a critical role in clinical presentation and symptom maintenance (18). Individuals with a tendency toward somatization may experience bodily sensations more intensely and report a wide array of somatic complaints, frequently seeking medical care for these symptoms.

In the context of FMS, somatization has been proposed as a psychological mechanism that not only contributes to symptom burden but may also interfere with accurate diagnosis and appropriate treatment (13, 19). Patients with FMS often display heightened sensitivity to interoceptive cues and an amplified focus on bodily discomfort, leading to disproportionate symptom reporting (20).

Epidemiologically, the prevalence of somatization varies globally, with estimates suggesting that up to 20% of primary care patients meet criteria for somatoform or related disorders (21). These findings underscore the importance of considering somatization not only as a general psychological phenomenon but also as a context-specific factor that may shape how FMS manifests across populations.

Alexithymia is a psychological trait characterized by difficulty in identifying and describing one’s emotions, as well as an externally oriented thinking style. It has been linked to a variety of medical conditions and is considered a transdiagnostic vulnerability factor associated with poorer health outcomes (22, 23). Studies have demonstrated elevated levels of alexithymia among individuals with FMS (24), with potential implications for both the onset and maintenance of somatic symptoms through impaired emotional processing (25, 26). This psychological dimension has been implicated in the complex interplay between emotional processing and somatic symptom expression, potentially contributing to the chronicity and perceived intensity of pain in FMS patients (27). Importantly, considering alexithymia solely as a deficit in emotion recognition or mood-related functioning may underestimate its broader relevance. The construct involves impairments across the entire affective system, influencing both physiological and cognitive-emotional integration (28). Given that alexithymic individuals tend to have limited emotional insight and heightened somatic focus, it is plausible that their bodily sensations may be perceived more intensely. In this context, somatosensory amplification—a tendency to experience normal bodily sensations as unusually intense and distressing—has emerged as a complementary construct that may interact with alexithymia to exacerbate FMS symptoms.

Somatosensory amplification refers to the tendency to experience bodily sensations as intense, harmful, and uncomfortable (29). This heightened awareness and exaggerated response to bodily sensations may intensify the level of symptoms in FMS, making the experienced pain more distressing and debilitating (30). The overlap between somatosensory amplification and alexithymia in individuals with FMS may indicate a complex psychophysiological loop. Indeed, previous research has indicated a significant association between alexithymia and somatosensory amplification in various psychosomatic disorders (31–33). This suggests that difficulty in expressing, identifying, or experiencing emotions could intensify bodily sensations, contributing to a cycle of pain and emotional distress by making emotional components even more elusive. Psychiatric comorbidity is frequently reported in fibromyalgia (34, 35), and the already reported presence of maladaptive coping or avoidance-oriented coping styles further serves as evidence of a psychological condition in FMS (27, 36).

Despite increased attention to the role of emotional traits in fibromyalgia, findings remain mixed and at times contradictory. Several studies have reported significant associations between alexithymia and clinical variables such as pain intensity, depression, anxiety, and somatosensory amplification (36–38). However, these associations frequently become statistically non-significant when negative affect is controlled for, suggesting that mood-related symptoms may account for the observed effects. Similarly, other studies have shown that depression, somatosensory amplification, and pain severity are predictive of functional impairment in FMS, whereas alexithymia and anxiety are not (39). Moreover, while alexithymia—especially its “difficulty identifying feelings” (DIF) subscale—has been found to correlate with anxiety, it has not consistently been associated with depression or pain-related variables (40). One study even reported that emotion regulation capacities and interoceptive sensitivity are associated with psychopathology in FMS, but not with alexithymia, further complicating the picture (27).

Conversely, other lines of research suggest that alexithymia may influence functionality indirectly. For instance, alexithymia has been found to predict physical impairment through the mediating effect of depression, indicating an indirect psychological pathway (41). In contrast, other studies report that DIF, as a core component of alexithymia, directly predicts functional impairment, while anxiety, depression, and other alexithymia dimensions are not significant contributors (42). Taken together, these inconsistencies underscore the need for more integrative statistical models and controlled comparisons to clarify the exact mechanisms linking emotional traits to functional outcomes in FMS. It has been shown that anxiety and depression explain medically unexplained physical symptoms, and that adding alexithymia to the model enhances its predictive capacity (28). In FMS patients, anxiety has also been shown to play a predictive role in functional somatic symptoms, rather than alexithymia, depression and somatic amplification (43). When anxiety and depression are controlled as covariates, the difference in alexithymia scores between the FMS and control groups is found to persist, however the effect is reported to be relatively reduced (36).

On the other hand, it has been observed that several of the aforementioned studies suffer from methodological limitations that reduce the generalizability of their findings. For example, some studies (28, 37, 39, 41) did not include a healthy control group, making it difficult to determine whether the psychological traits observed are unique to FMS or reflect general psychological distress. In addition, studies that did incorporate comparisons often relied on relatively small sample sizes (e.g., n < 50 per group), which may have limited statistical power and increased the likelihood of Type II errors (27, 40, 43, 44). Beyond these methodological issues, the literature presents clearly conflicting results: while some findings suggest that alexithymia and anxiety are not significant predictors of functional impairment, others report both direct and indirect pathways linking these emotional traits to disease outcomes. These inconsistencies, along with limited study designs, highlight the need for a more comprehensive evaluation using larger samples, healthy comparison groups, and mediation-based statistical models to clarify the psychological mechanisms underlying FMS.

Taken together, these methodological limitations and inconsistencies in prior findings highlight the need for further clarification regarding the psychological mechanisms contributing to fibromyalgia. The current study was designed to address these gaps by examining both group differences and mediation-based relationships involving key emotional variables. Specifically, we aimed to compare levels of alexithymia, anxiety, depression, and somatosensory amplification between female individuals with FMS and healthy controls. Furthermore, we conducted mediation analyses within the FMS group to investigate whether anxiety and depression mediate the relationships between (a) alexithymia and functional impairment, and (b) somatosensory amplification and functional impairment. In addition, we sought to explore whether anxiety and depression account for the group differences observed in somatosensory amplification and alexithymia levels, and whether the presence of alexithymia corresponds to distinct symptom severity profiles. To this end, we planned multivariate and univariate analyses of covariance to test these relationships in a more controlled framework. By combining group-level comparisons with structural psychological modeling, this study seeks to provide a more integrative perspective on the emotional and somatic processes underlying functional limitations in FMS. Clarifying these associations may inform future biopsychosocial intervention strategies and improve treatment outcomes in this challenging patient population.

Based on the literature and theoretical framework outlined above, we formulated the following hypotheses: (1) Individuals with fibromyalgia will exhibit significantly higher levels of alexithymia (particularly the DIF subdimension), somatosensory amplification, anxiety, and depression compared to healthy controls. (2) Within the FMS group, higher levels of alexithymia and somatosensory amplification will be significantly associated with greater functional impairment. (3) Anxiety and depression will mediate the relationship between (a) difficulty identifying feelings (DIF) and functional impairment, and (b) somatosensory amplification and functional impairment. (4) The differences in somatosensory amplification and alexithymia between the FMS and control groups will be attenuated when anxiety and depression are statistically controlled.

## 2 Materials and methods

### 2.1 Participants

The study involved 142 adult female patients (Mean age = 32.197 ± 4.413) diagnosed with FMS. These patients initially sought care at physical therapy and rehabilitation as well as rheumatology outpatient clinics between January to March 2021. They were subsequently referred to the psychiatry unit for participation in the study. All patients had been assessed by a physical therapy and rehabilitation specialist and met the American College of Rheumatology criteria for FMS (15, 45). Data for the healthy control group were collected using a convenience sampling method with purposive demographic matching to the patient group. Although initial recruitment began through voluntary respondents, subsequent participants were selected to match the FMS group on key sociodemographic variables such as age, marital status, and employment status.

The control group consisted of 141 women with a mean age of 31.48 years (SD = 3.58), matched with the FMS group in terms of age, *t* (281) = -1.496, *p* = .136, marital status, *χ²*(1, N = 283) = 0.77, *p* = .086, and working status, *χ²*(1, N = 283) = .001, *p* = .978. This sampling strategy was adopted to increase comparability between groups while accounting for key demographic variables.

The study sample consisted entirely of women, based on the substantially higher prevalence of fibromyalgia among females, with a reported female-to-male ratio of approximately 9:1 (46). This sampling approach was also adopted to reduce the potential confounding effects of gender on pain perception and emotional processing. The inclusion criteria for the study were being a female aged between 18 and 45 years and having a diagnosis of fibromyalgia, in order to minimize the potential confounding effects of hormonal changes associated with menopause (47). The exclusion criteria included cognitive impairment, a primary psychotic disorder, or a somatoform disorder, as defined by DSM-5 criteria. In order to minimize the influence of prolonged symptom exposure and associated psychological adaptations, participants who had been diagnosed with fibromyalgia more than 5 years prior were excluded. Additionally, participants who had received pharmacological treatment for fibromyalgia within the past 6 months were excluded, as medications may attenuate symptom severity and confound the assessment of psychological factors.

### 2.2 Measures

#### 2.2.1 Sociodemographic Data Form

This form was designed by researchers to record the participants’ social and demographic data, including age, marital status, employment status, level of education, and history of chronic physical or psychiatric illness.

#### 2.2.2 Beck Depression Inventory (BDI)

The Beck Depression Inventory is a 21-item self-report questionnaire that assesses the severity of depression (48). Individuals are asked to rate themselves on each item on a 0–3 scale (0: least, 3: most; score range: 0 to 63). The total score is the sum of all items. Increasing scores indicate the severity and intensity of depressive symptoms. The Turkish version of the scale has been shown to be valid and reliable; internal consistency (Cronbach’s Alpha) was reported as 0.89, and test-retest reliability was 0.81 (49). In the current study, Cronbach’s alpha was good (α FMS = .86; α control = .91).

#### 2.2.3 Beck Anxiety Inventory (BAI)

The Beck Anxiety Inventory is a 21-item self-report questionnaire (50) used to measure the subjective and physiological symptoms of anxiety. Participants rate each item on a 4-point Likert scale (0: not at all, 3: severely, score range: 0 to 63). Increasing scores indicate the severity of anxiety. The Turkish version of the scale has been shown to be a valid and reliable measurement tool. The internal consistency (Cronbach’s Alpha) of the Turkish version was found to be 0.93, and test-retest reliability was 0.83 (51). The BAI demonstrated good reliability in this study (α FMS = .89; α control = .93).

#### 2.2.4 Toronto Alexithymia Scale (TAS-20)

The 20-Item Toronto Alexithymia Scale measures alexithymia. This is a self-administered questionnaire consisting of 20 items scored on a 5-point Likert scale recording respondents degree of agreement/disagreement for each statement (1 = I don’t agree at all; 2 = I don’t agree very much; 3 = I’m not either neither agree nor disagree; 4 = I agree in part; 5 = I completely agree). TAS-20 has three subscales representing three main facets of alexithymia: the Difficulty Identifying Feelings (DIF, seven items) subscale, measures difficulty in distinguishing between specific emotions and/or bodily sensations related to emotional arousal; the Difficulty Describing Feelings (DDF, five items) subscale, indicates inability to verbalize one’s experienced emotions; the Externally Oriented Thinking (EOT, eight items) subscale, indicates the tendency to focus attention externally instead of considering inner emotional experience. It is the most widely utilized instrument in both clinical and research settings for the assessment of alexithymia, demonstrating satisfactory internal consistency (Cronbach’s alpha = 0.81) and robust test–retest reliability (0.77, p < 0.01) (52). The TAS-20 scale has been found to be valid and reliable in the Turkish population. In examining the internal consistency of the scale and its subscales, the Cronbach’s alpha values were calculated as follows: α = 0.78 for the total scale, α = 0.80 for Factor 1, α = 0.57 for Factor 2, and α = 0.63 for Factor 3 (53). TAS-20 demonstrated good internal consistency in the current study (α FMS = .81; α control = .77).

#### 2.2.5 Somatosensory Amplification Scale (SSAS)

The Somatosensory Amplification Scale (SSAS), developed by Barsky et al. (29), is a 10-item measure that uses a five-point Likert scale to investigate an individual’s tendency for somatosensory amplification, defined as the tendency to experience bodily sensations as intense, uncomfortable, and often painful. Participants are asked to rate 10 statements on a 5-point scale (1: not at all, 5: extremely), reflecting how accurately these statements generally describe their experiences. A higher score signifies a stronger inclination towards somatosensory amplification. The validity and reliability of the Turkish version were established by Güleç and Sayar, with a test–retest reliability coefficient of r = 0.73 and internal consistency values ranging from α = 0.62 to 0.76 (54). In this study, SSAS showed good internal consistency (α FMS = .79; α control = .72).

#### 2.2.6 Fibromyalgia Impact Questionnaire (FIQ)

The instrument consists of 10 self-report items designed to evaluate the condition, progression, and treatment outcomes of individuals with fibromyalgia 25. It is brief, taking approximately five minutes to complete, and involves the participant rating their experiences. The scale assesses multiple domains including general well-being, pain, fatigue, work disability, functional difficulty, stiffness, morning fatigue, anxiety, and depression. The first item comprises 11 questions rated on a 4-point Likert scale focusing on physical function. Items 2 and 3 evaluate the number of days the patient felt well and the number of days they were unable to work due to fibromyalgia symptoms. Items 4 through 10 address various aspects such as work difficulty, morning fatigue, stiffness, anxiety, depression, pain, and fatigue. Each domain is scored from 0 (no impairment) to 10 (severe impairment), with a total score ranging from 0 to 100. Lower total scores indicate a lesser impact of fibromyalgia (55). The Turkish version of the scale has been shown to be a valid and reliable measurement tool. The internal consistency (Cronbach’s Alpha) of the Turkish version was found to be 0.81, and test-retest reliability was 0.72 (56). The FIQ demonstrated adequate reliability in this study (α FMS = .71).

### 2.3 Procedures

The data were collected in the rest room of a psychiatric outpatient clinic. We used the Sociodemographic Data Form to ascertain the individuals’ demographic and individual characteristics. The Beck Depression Inventory (BDI) was employed to assess their depressive symptom severity, and the Beck Anxiety Inventory (BAI) was used to assess their anxiety levels. Additionally, the Toronto Alexithymia Scale (TAS-20) evaluated their alexithymia levels, and the Somatosensory Amplification Scale (SSAS) measured their tendency for somatosensory amplification. The Fibromyalgia Impact Questionnaire (FIQ) was utilized to assess the severity of their fibromyalgia symptoms.

All participants provided written informed consent before beginning the study. The scales were administered in a randomized order to minimize potential order effects, such as response fatigue or priming bias. The study was approved by the ethics committee of the local university.

### 2.4 Statistical analyses

Before starting the analyses, the data were examined for univariate outliers. The data of two individuals from the FMS group and three individuals from the healthy control group, which were identified as outliers, were excluded from the analysis. Data normality was assessed using kurtosis (−0.504 to 0.956) and skewness (−0.794 to 1.168) values. Differences between groups, relationships between variables, and analyses for regression were conducted using the IBM SPSS 25.0 (Statistical Package for the Social Sciences) software package.

First, differences between FMS patients and the healthy control group in terms of BAI, BDI, SSAS, and TAS-20 scores were examined. In line with this purpose, Multivariate Analysis of Variance (MANOVA) was conducted, and to reduce the risk of Type I error in multiple comparisons, p=0.008 was set (i.e. Bonferroni-corrected, for six comparisons). To explore the potential effect of anxiety and depression scores on group differences in SSAS and TAS scores, a multivariate analysis of covariance (MANCOVA) was conducted, with anxiety and depression scores included as covariates. In the MANCOVA analysis, p=0.012 was set (i.e. Bonferroni-corrected, for four comparisons). Then, a MANOVA analysis was conducted to examine whether there were differences in anxiety, depression, and SSAS scores between those with and without alexithymia based on TAS-20 cut-off scores (p = 0.017, for three comparisons). To explore the potential effect of anxiety and depression scores on group differences in SSAS score, Analysis of Covariance (ANCOVA) was conducted, with anxiety and depression scores included as covariates. While considering the assumptions of linearity, homogeneity of variances and covariances, and absence of multicollinearity for MANOVA; for MANCOVA and ANCOVA assumptions of linearity, homogeneity of variances and covariances, absence of multicollinearity and homogeneity of regression slopes were considered. However, since the assumption of homogeneity of variance was not initially met for the ANCOVA, Hartley’s Fmax test was employed to further examine variance homogeneity, and according to this test, the homogeneity assumption was found to be met.

The chi-square test was employed to examine relationships among qualitative data. The Pearson correlation coefficient was calculated among the scores of BAI, BDI, TAS-20, SSAS and FIQ.

The mediating roles of anxiety and depression in the relationship between difficulty identifying feelings, somatic amplification, and fibromyalgia-related functional impairment were tested using two separate mediation models. Mediation analysis was performed using the jAMM package within the JAMOVI 2.3.28 software, a robust statistics program (57).

## 3 Results

### 3.1 Group differences

The MANOVA results, which examined the TAS subscales, BAI, BDI, and SSAS scores between the groups, revealed a significant multivariate group effect, *V*=0.346, *F* [6, 276] = 24.326, *p* < 0.001, *ηp^2^* = 0.346. **Table 1** displays the MANOVA results and descriptive statistics. For TAS subscale scores, the DIF score was higher in the FMS group compared to healthy controls, while there was no significant difference between groups in DDF and EOT scores. BDI and BAI scores were higher in FMS patients vs. healthy control. The FMS group also had higher scores on SSAS than healthy controls. Examining effect sizes, the effect for BAI is markedly greater, followed by DIF, SSAS and BDI respectively.

**Table 1:** Descriptive statistics, MANOVA and MANCOVA results for comparing FMS and healthy control groups.

Variable	FMS Group	Healthy Control	Group Effect (without covariate)			Group Effect (with covariate)		
	$\bar{X}$ ± SD	$\bar{X}$ ± SD	F	P	$\eta_{p}^{2}$	F	p	$\eta_{p}^{2}$
DIF	20.589 ± 4.800	15.61 ± 5.56	65.023	0.000	0.188	19.391	0.000	0.065
DDF	13.347 ± 4.046	12.401 ± 4.018	3.896	0.049	0.014	4.266	0.040	0.015
EOT	21.105 ± 4.575	21.319 ± 4.707	0.150	0.699	0.001	1.497	0.222	0.005
SSAS	33.552 ± 7.678	27.750 ± 7.590	40.863	0.000	0.127	5.301	0.022	0.019
BAI	28.993 ± 11.598	15.390 ± 14.185	78.033	0.000	0.217			
BDI	21.605 ± 8.205	14.051 ± 13.602	32.052	0.000	0.102			

DIF, TAS-20 Difficulty Identifying Feelings subscale; DDF, TAS-20 Difficulty Describing Feelings subscale; TAS-20 EOT, Externally Oriented Thinking subscale; FIQ, Fibromyalgia Impact Questionnaire total score; SSAS, Somatosensory Amplification Scale total score; BAI, Beck Anxiety Inventory total score; BDI, Beck Depression Inventory total score; Group Effect: Fibromyalgia Group vs. Healthy Control Group.

Based on the commonly accepted cut-off scores for the BAI (≥16 indicating at least moderate anxiety; Beck et al., 1988) and BDI (≥20 indicating at least moderate depression; Beck et al., 1996), it was observed that 63.3% (n = 179) of all participants reported moderate to severe anxiety symptoms, and 58% (n = 164) reported moderate to severe depressive symptoms. When broken down by group, 81.7% (n = 116) of the FMS patients and 44.7% (n = 63) of the controls met the threshold for moderate to severe anxiety. Similarly, 78.9% (n = 112) of the FMS group and 36.9% (n = 52) of the control group reported moderate to severe depressive symptoms. These figures highlight the markedly higher emotional distress in the FMS group.

MANCOVA was performed to control for the influence of anxiety and depression on between-group differences in psychological variables. The analysis showed a significant overall group effect even after accounting for anxiety and depression levels (Wilks’ Λ = 0.84, F [4, 276] = 13.35, p <.001, ηp² = 0.16). *Post hoc* tests indicated that the between-group difference in difficulty identifying feelings (DIF) scores remained significant, although the effect size was substantially reduced. In contrast, the group difference in SSAS scores became non-significant. No significant differences were found for the DDF and EOT subscales. (See **Table 1**).

In the MANOVA examining BAI, BDI, and SSAS scores among individuals with and without alexithymia according to the TAS-20 cutoff score, the multivariate group effect was significant, *Wilks’ Λ* = 0.776, *F* <(>3, 279<)> = 26.804, *p* < 0.0001, *ηp²* = 0.224. **Table 2** represents the MANOVA results and descriptive statistics. Individuals with alexithymia had higher BAI, BDI, and SSAS scores than those without alexithymia (*p* < 0.017). To examine the covariate effect of anxiety and depression in alexithymic and non-alexithymic individuals, an ANCOVA analysis was conducted with SSAS scores as the dependent variable. The analysis indicated that the main effect was significant, F (3, 279) = 40.207, p < 0.001, ηp² = 0.302. Among the covariates, the effect of anxiety was significant, F (1, 281) = 74.901, p < 0.001, ηp² = 0.212, whereas the effect of depression was found to be non-significant, F (1, 281) = 0.168, p = 0.682, ηp² = 0.001. Although the MANOVA revealed a significant group difference in SSAS scores between alexithymic and non-alexithymic individuals, this difference was no longer statistically significant after controlling for anxiety and depression in the ANCOVA, F (1, 281) = 0.938, p = 0.334, ηp² = 0.003.

**Table 2:** Descriptive statistics and MANOVA results for comparing alexithymic and non-alexithymic groups.

Variable	Alexithymic	Non-alexithymic	Group Effect		
	$\bar{X}$ ± SD	$\bar{X}$ ± S	F	P	$\eta_{p}^{2}$
BAI	31.382 ± 11.499	19.316 ± 14.314	40.095	0.000	0.125
BDI	27.529 ± 9.375	14.777 ± 10.839	76.060	0.000	0.213
SSAS	32.863 ± 8.163	29.965 ± 8.048	6.652	0.010	0.023

DIF, TAS-20 Difficulty Identifying Feelings subscale; DDF, TAS-20 Difficulty Describing Feelings subscale; TAS-20 EOT, Externally Oriented Thinking subscale; FIQ, Fibromyalgia Impact Questionnaire total score; SSAS, Somatosensory Amplification Scale total score; BAI, Beck Anxiety Inventory total score; BDI, Beck Depression Inventory total score; Group Effect: Fibromyalgia Group vs. Healthy Control Group.

### 3.2 Relationships between anxiety, depression, functional impairment, and somatic amplification in FMS

The correlation coefficients between TAS-20 subscales, BAI, BDI, FIQ, and SSAS scores are presented in **Table 3** for the FMS group to examine the relationships among these variables in individuals with FMS. Coefficients generally ranged from weak to strong. The correlations of BDI and BAI with DIF, DDF, SSAS, and FIQ ranged from medium to strong. The relationships of FIQ with DIF, DDF, and SSAS were between medium to large range. SSAS showed a moderate correlation with DDF, a non-significant correlation with DIF, and generally non-significant correlations with other scales for EOT.

**Table 3:** Pearson correlation coefficients between TAS-20, SSAS, FIQ, BAI and BDI scores in FMS group.

Measure	DIF	DDF	EOT	SSAS	FIQ	BAI	BDI
1. DIF	–						
2. DDF	.461***	–					
3. EOT	.215**	.230**	–				
4. SSAS	.137	.326***	.013	–			
5. FIQ	.365***	.305***	.037	.377***	–		
6. BAI	.335***	.361***	.006	.473***	.628***	–	
7. BDI	.521***	.358***	-.092	.248**	.439***	.589***	–

**p < .01; ***p < .001. n= 142. DIF, TAS-20 Difficulty Identifying Feelings subscale; DDF, TAS-20 Difficulty Describing Feelings subscale; TAS-20 EOT, Externally Oriented Thinking subscale; FIQ, Fibromyalgia Impact Questionnaire total score; SSAS, Somatosensory Amplification Scale total score; BAI, Beck Anxiety Inventory total score; BDI, Beck Depression Inventory total score.

Considering the between-group differences, covariate effects, and the relationships between anxiety, depression, and other clinical variables, mediation analyses were conducted to examine the potential mediating roles of anxiety and depression in the relationships among difficulty identifying feelings, functional impairment, and somatic amplification in the FMS group. Anxiety and depression were tested as separate mediators to assess their independent effects (see **Table 4**).

**Table 4:** Mediation analysis results.

Mediator: Anxiety								
				95% C.I. (a)				
Type	Effect	Estimate	SE	Lower	Upper	β	z	p
Indirect	DIF⇒ BAI ⇒ FIQ	0.534	0.158	0.224	0.844	0.143	3.38	< .001
	SSAS⇒ BAI ⇒ FIQ	0.526	0.115	0.300	0.752	0.225	4.57	< .001
Component	DIF ⇒ BAI	0.667	0.171	0.331	1.003	0.276	3.89	< .001
	BAI ⇒ FIQ	0.801	0.117	0.572	1.030	0.518	6.85	< .001
	SSAS ⇒ BAI	0.657	0.107	0.447	0.867	0.435	6.13	< .001
Direct	DIF ⇒ FIQ	0.661	0.251	0.168	1.153	0.177	2.63	0.009
	SASS ⇒ FIQ	0.254	0.168	-0.075	0.583	0.109	1.51	0.131
Total	DIF ⇒ FIQ	1.195	0.277	0.652	1.737	0.319	4.32	< .001
	SSAS ⇒ FIQ	0.780	0.173	0.441	1.119	0.334	4.51	< .001
Mediator: Depression								
				95% C.I. (a)				
Type	Effect	Estimate	SE	Lower	Upper	β	z	p
Indirect	DIF ⇒ BDI ⇒ FIQ	0.505	0.172	0.166	0.843	0.135	2.93	0.003
	SSAS⇒BDI ⇒FIQ	0.115	0.057	0.002	0.227	0.049	2.00	0.046
Component	DIF ⇒ BDI	0.848	0.121	0.611	1.085	0.496	7.02	< .001
	BDI ⇒ FIQ	0.595	0.185	0.233	0.957	0.272	3.22	0.001
	SSAS ⇒ BDI	0.193	0.076	0.045	0.341	0.180	2.55	0.011
Direct	DIF ⇒ FIQ	0.690	0.309	0.084	1.295	0.184	2.23	0.026
	SSAS ⇒ FIQ	0.666	0.170	0.332	0.999	0.285	3.91	< .001
Total	DIF ⇒ FIQ	1.195	0.277	0.652	1.737	0.319	4.32	< .001
	SSAS ⇒ FIQ	0.780	0.173	0.441	1.119	0.334	4.51	< .001

DIF, TAS-20 Difficulty Identifying Feelings subscale; DDF, TAS-20 Difficulty Describing Feelings subscale; TAS-20 EOT, Externally Oriented Thinking subscale; FIQ, Fibromyalgia Impact Questionnaire total score; SSAS, Somatosensory Amplification Scale total score; BAI, Beck Anxiety Inventory total score; BDI, Beck Depression Inventory total score.

**Figure 1:**
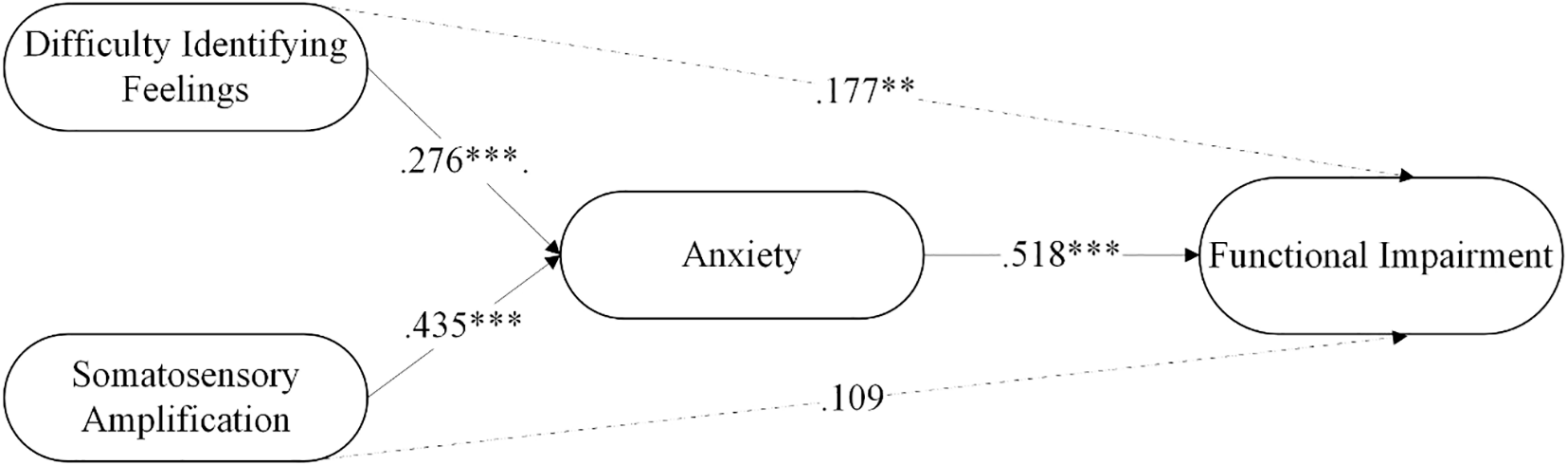
The mediating role of anxiety in the relationship between difficulty identifying feelings and functional impairment in FMS patients. **p < .01; ***p < .001.

As seen in **Table 4**, difficulty identifying feelings significantly predicted functional impairment through the mediation of anxiety, with anxiety partially mediating the relationship between difficulty identifying feelings and functional impairment. Somatic amplification significantly predicted functional impairment through the mediation of anxiety, with anxiety fully mediating the relationship between somatic amplification and functional impairment. Moreover, the model explained 29.82% of the variance in anxiety and 43.1% in functional impairment. **Figure 1** summarizes the results of the mediation analysis.

**Figure 2:**
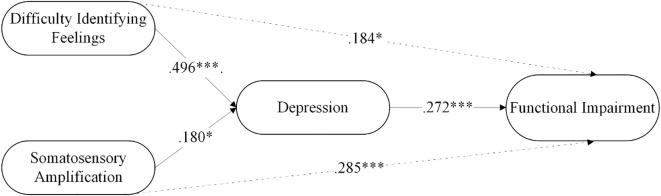
The mediating role of depression in the relationship between somatosensory amplification and functional impairment in FMS patients. *p < .05, ***p < .001.

The findings related to the mediation of depression are presented in **Table 4**. Difficulty identifying feelings significantly predicted functional impairment through the mediation of depression, with depression partially mediating the relationship between difficulty identifying feelings and functional impairment. Similarly, somatic amplification significantly predicted functional impairment through the mediation of depression, with depression partially mediating the relationship between somatic amplification and functional impairment. The model explained 24.3% of the variance in depression and 30.3% in functional impairment. The mediation analysis is summarized in **Figure 2**.

## 4 Discussion

This study provides significant insights into the psychological and somatic characteristics of individuals with FMS, highlighting distinct differences between the FMS group and healthy controls.

Our findings demonstrated that individuals with FMS exhibit significantly higher levels of anxiety, depression, and somatosensory amplification, and particularly experience challenges in identifying feelings in the aspect of alexithymia. However, when the effects of anxiety and depression were statistically controlled, only DIF remained significant between the groups, and its effect size also decreased. In the literature, it has also been reported that alexithymia scores – DIF and DDF (36, 58) or DIF, DDF, and EOT (40) subscale scores, as well as the total alexithymia score (27, 43, 59) are significantly higher in individuals with FMS compared to healthy controls. Additionally, in individuals with FMS, psychological symptoms such as anxiety and depression, or negative affect (27), have been found to be significantly elevated compared to the control group (36, 40, 59). Furthermore, heightened interoceptive sensitivity (Schmitz et al., 2021) and increased somatic amplification (43) have also been observed in these patients.

Findings in literature are parallel to our study in various aspects. However, in our study, a significant difference was found only in the DIF score on the TAS-20 scale. Due to the application of the Bonferroni correction, the DDF score, although close to the significance threshold, was not statistically significant. This correction has actually made the significance levels statistically more reliable. Indeed, some studies comparing TAS-20 subscale scores do not report statistical findings related to this correction (27, 40, 58). Additionally, it has been observed that in some of the aforementioned studies comparing FMS patients and control groups, the sample sizes were relatively small (27, 36, 40, 43, 58). Moreover, it has been identified that several studies comparing alexithymia levels in FMS and control groups did not control for psychological symptoms such as anxiety and depression as covariates when interpreting group differences (27, 40, 43). However, Montoro et al. (36), in contrast to these studies and consistent with the current study, conducted such an analysis and reported that the effect size associated with group differences in alexithymia scores diminished after adjusting for anxiety and depression. On the other hand, the relationship between alexithymia and anxiety, depression, or emotional disorders is well-documented (60–62). It has been stated that this relationship can be bidirectional, where alexithymia may emerge as a result of emotional problems, exacerbating the symptoms, or as a stable personality trait leading to psychological symptoms (62). Additionally, consistent with the findings of the current study, there is also evidence suggesting that difficulty in identifying feelings is the strongest unique predictor of psychopathology (61); and somatic amplification is reported to be closely link psychological conditions (31, 63). In summary, it appears that individuals with FMS may experience dysfunction in identifying feelings related to alexithymia and heightened somatic amplification, however psychiatric symptoms play a significant role on both alexithymic traits and somatic amplification.

Another important finding of our study is that depression, anxiety, and somatic amplification scores were higher among alexithymic individuals compared to non-alexithymic individuals. However, when anxiety and depression were included as covariates, the previously significant difference in somatic amplification scores between groups was no longer statistically significant. While the relationship between alexithymia and psychopathology mentioned earlier was evident, the effect of alexithymia on somatic amplification appears to be primarily related to psychological symptoms such as anxiety and depression. In other words, somatic amplification may be more closely associated with co-occurring psychological problems rather than directly linked to alexithymia. In parallel, a recent latent profile analysis study found that somatic amplification is strongly associated with anxiety and depression in the context of pain and other comorbid disorders (64). Additionally, it has been reported that depression and anxiety are primary determinants of both alexithymia and somatic amplification in chronic pain patients (31).

In our study, the significant relationships found in the FMS group between alexithymia (particularly DIF and DDF), depression, anxiety, somatic amplification, and functional impairment are consistent with the findings in the literature. Previous studies have also shown relationships between alexithymia (DIF and/or DDF) and depression, anxiety, psychological symptoms, health-related quality of life, pain experiences, functional impairment, pain intensity, or somatic amplification in various chronic pain conditions, such as FMS (36, 39–43).

Moreover, of particular interest in our study, the relationship between DIF, somatic amplification, and functional impairment in the FMS group was found to be mediated by both anxiety and depression in two separate models. In both models, the mediation was partial, indicating that while DIF and somatic amplification had some direct effects on functional impairment, a significant proportion of these effects was transmitted through anxiety and depression. These findings align with a biopsychosocial model of chronic pain, which posits that the interaction between psychological vulnerability (e.g., alexithymia), maladaptive physiological processing (e.g., somatic amplification), and emotional distress (e.g., anxiety and depression) contributes to the experience and maintenance of chronic pain and disability. In this framework, psychological traits such as alexithymia may serve as predisposing factors (diatheses), while emotional dysregulation and stress-related psychopathology act as mediators that exacerbate functional impairment.

These findings are consistent with previous studies indicating that heightened somatic sensitivity and emotional dysregulation exacerbate the functional limitations experienced by FMS patients (20, 38, 41). Tesio et al. (41) found that anxiety and depression mediated the relationship between DIF and health-related quality of life. In patients with chronic pain, the relationship between alexithymia and pain is commonly reported to be mediated by negative affect or distress (20, 38). Additionally, it has been noted that in chronic conditions, pain increases both the recurrence of psychological issues and the severity of anxiety and depression (65, 66). Findings related to maladaptive coping or emotional dysregulation in chronic pain conditions and their relationship with psychological distress (27, 36) also point to the relationship established with emotions, coping difficulties, and their impact on psychiatric outcomes. Furthermore, depression and anxiety have been reported to be associated with pain-related disability and impaired quality of life (67).

In summary, the study findings indicate that DIF in the context of alexithymia and somatosensory amplification are clinically significant in FMS; however, anxiety and depression were found to play a crucial role in these conditions. Indeed, the results of the mediation analysis also support the significant predictive role of emotional disturbances. In clinical assessment and interventions in FMS patients, increasing emotional awareness and preventing catastrophic interpretation of somatic sensations seem important. Given their mediating role in the relationship between emotional processing difficulties and functional impairment, co-occurring anxiety and depression should be prioritized as key treatment targets in clinical interventions for FMS. Encouraging active coping strategies may also be supportive in this context. Indeed, in the literature, it is noted that fear and anxiety related to pain contribute to disability through passive or avoidance-oriented pain coping behaviors (68), that these factors also lead to physical deconditioning, pain, and psychological problems, and that catastrophizing tendencies influence both pain and physical disability (69–72). In parallel, findings in the literature indicate that interventions such as Cognitive Behavioral Therapy (CBT) in chronic pain groups are effective in reducing somatosensory amplification (73), decreasing pain intensity and enhancing emotional well-being (74), reducing symptoms of anxiety and depression, improving pain management, increasing functionality (43), improving alexithymic tendencies (75), and addressing the emotional and affective dimensions of chronic pain treatment (76). Therefore, promoting active coping strategies (e.g., reducing avoidance-oriented behaviors) and implementing interventions to support emotional regulation can help develop the tendency to recognize and process emotions, maintain bodily sensations and pain perception within functional limits, and positively impact psychological well-being. Consequently, improvements in physical and psychological functionality — including daily activity performance, pain coping, and emotional well-being — could also be achievable in patients with Fibromyalgia Syndrome (FMS) through interventions that enhance emotional regulation and reduce somatic distress.

Beyond replicating existing findings in a larger and more methodologically robust sample, this study offers several novel contributions to the literature on FMS. First, by concurrently examining alexithymia, somatosensory amplification, anxiety, and depression within a mediation framework, it provides a more integrated understanding of how emotional dysregulation may contribute to functional impairment. Specifically, the identification of anxiety and depression as mediators between difficulty identifying feelings (DIF), somatosensory amplification (SSAS), and FMS-related dysfunction offers a nuanced explanation for the interplay of psychological and somatic processes. Furthermore, by isolating the DIF subcomponent of alexithymia as particularly relevant, our findings underscore the importance of targeting emotional awareness in clinical interventions—an area that has received limited empirical attention in FMS populations.

While our findings contribute to the understanding of psychological and somatic factors in FMS, certain limitations should be considered. First, the cross-sectional nature of the study limits the ability to infer causal relationships between the variables. Longitudinal studies are needed to clarify the temporal dynamics of anxiety, depression, somatosensory amplification, and alexithymia in FMS. Additionally, the reliance on self-report measures may introduce biases related to participants’ subjective experiences, and future research could benefit from integrating objective physiological measures of pain and emotional processing. Also, educational level and socioeconomic status (SES) were not formally controlled between groups. Although participants were matched on age, marital status, and employment status, unmeasured demographic factors such as education and SES may have influenced symptom expression or access to care, and this should be considered when interpreting the generalizability of the results. Future studies should also explore the potential benefits of psychological interventions targeting both difficulty in identifying feelings and somatosensory amplification, examining their effects on anxiety and depression, and how these improvements may reflect on functionality in FMS patients. Cognitive Behavioral Therapy (CBT), mindfulness-based interventions, and emotional regulation strategies may offer promising approaches for reducing the psychological and somatic burden in this patient group, ultimately enhancing their quality of life.

## 5 Conclusion

In conclusion, this study highlights the significant role of DIF and somatosensory amplification in the experience of FMS and has particularly clarified the impact of anxiety and depression on these variables, as well as the role of psychological conditions in functionality. Our findings underscore the importance of addressing both the psychological and somatic aspects of FMS to provide comprehensive care. Through a biopsychosocial, holistic approach in both assessment and treatment, healthcare providers may be better equipped to address the complex symptomatology of fibromyalgia. Future interventional research is needed to evaluate whether targeting sensory processing abnormalities and emotional dysregulation can lead to improvements in clinical outcomes.

## Data Availability

The raw data supporting the conclusions of this article will be made available by the authors, without undue reservation.
